# Does cancer deserve special treatment when health technologies are prioritized?

**DOI:** 10.1186/2045-4015-2-45

**Published:** 2013-11-18

**Authors:** Paul Hansen

**Affiliations:** 1 Department of Economics, University of Otago, Dunedin 9054, New Zealand

## Abstract

Despite most new cancer treatments having relatively high costs and low health benefits, they are often funded ahead of treatments for other illnesses. And yet, according to the article by Dan Greenberg and colleagues, most Israeli oncologists and family physicians think that new cancer treatments should not receive such a high priority and that cost-effectiveness data should be used to support funding decisions. In this commentary, I point out that the increasing pressure worldwide when prioritizing health technologies to widen the scope of the benefits that are recognized beyond just narrowly-defined health benefits would almost certainly include the special characteristics of cancer. Future research would be worthwhile into how the criteria for prioritizing technologies should be incorporated into prioritization frameworks in practice, including, in particular, how to resolve the inherent trade-offs.

This is a commentary on http://www.ijhpr.org/content/2/2/44/

## Commentary

The article by Dan Greenberg and colleagues reports on a national survey of Israeli oncologists and family physicians into their views on patient access and coverage decisions with respect to interventions for cancer and congestive heart failure (CHF) respectively [1]. Among other things, the survey probed whether these two groups of physicians regard cancer treatments as being of higher value, and so of higher priority, than treatments for other medical conditions – the latter, in effect, represented by CHF (like cancer, a life-threatening condition). Such a ‘cancer premium’ has been found in similar surveys in the US and Canada, and has been inferred from the recommendations of organizations responsible for prioritizing health technologies in several countries, including Israel’s Public National Advisory Committee (also known as ‘the Basket Committee’).

Why might cancer treatments be accorded special status relative to other treatments? Given that cancer is often fatal, many cancer interventions are, in effect, ‘end-of-life’ treatments; and, correspondingly, cancer sufferers are often also in greater need than other patients (albeit cancer patients’ potential health gains from treatment are almost always inevitably less). There are also undoubtedly significant psychological and cultural aspects too arising from cancer’s fearsome reputation and associated taboos [2].

Interestingly, the survey reported on in the article reveals that Israeli oncologists and family physicians, on the whole, do *not* favor interventions for cancer over ones for other illnesses. Most respondents think that cancer (and also CHF) treatments should not receive a higher priority, all else being equal. As well as being at variance with the above-mentioned international evidence, this finding challenges the Basket Committee’s apparent favoritism, according to the authors, towards cancer treatments.

The controversy internationally surrounding the funding of new cancer treatments (see the references in the original article) derives from the stylized fact that most new cancer treatments have very high costs and low health benefits relative to treatments for other disease groups. Some cost tens of thousands of dollars per patient (sometimes in excess of $US100,000, such as for the drugs Vandetanib and Vismodegib mentioned in the article) but delay death by only months (even weeks). And yet many high cost cancer treatments do get funded in Israel and other countries.

In light of the above-mentioned controversy, I find it reassuring that a large majority (76%) of the surveyed physicians think that cost-effectiveness data should be used to support decisions about whether to include cancer and CHF treatments – and, presumably, other technologies too – in Israel’s health basket. It is worthwhile emphasizing that this finding is in the context of, according to the authors, the Basket Committee not explicitly using the results from cost-effectiveness analysis (CEA), although “studies have suggested that in many cases the recommended technologies … are those with favorable cost-effectiveness results”.

As a ‘health’ economist, in particular one from New Zealand, it intrigues me that there continue to be developed countries, including Israel (and the US [3]), in which formal CEA is not explicitly an important input into health technology prioritization. In New Zealand – and many other countries too [4] – CEA, specifically cost-utility analysis, is a major component (but not the only one) of the decision-making process followed by the Pharmaceutical Management Agency (PHARMAC) [5].

The defining characteristic of such economics-based frameworks – in contrast to non-economics-based frameworks (e.g. ‘needs assessment’) – is that the outcomes of alternative funding allocations are compared relative to each other, with the objective of maximizing the value of the various benefits realized from the money spent. It seems that most respondents to the survey, as well as the Basket Committee, subscribe to this over-arching ‘philosophy’ – one which is probably common sense to most people, being qualitatively similar to how a household might think about allocating its budget when doing the weekly grocery shopping, for example.

Where the possible confusion and debate arises, however, is with respect to how the various benefits realizable from the competing health technologies are defined and explicitly recognized. There is increasing pressure worldwide to widen their scope beyond just narrowly-defined health benefits such as are commonly represented using Quality-Adjusted Life Years [6]. In the present context, these wider benefits would almost certainly include the special characteristics of cancer mentioned earlier (and perhaps others). The challenge in general is how to incorporate these wider benefits into health technology prioritization frameworks in practice, in a systematic and transparent fashion that can also be easily communicated to stakeholders, including the general public [7].

Strangely, despite the above-mentioned ringing endorsement from Israeli physicians of the use of cost-effectiveness data to support funding decisions, 65% of oncologists and 55% of family physicians also agreed (some strongly) with this statement in the survey: “Every patient in Israel should have access to effective cancer treatments regardless of their cost”. (For CHF the corresponding percentages were 73% and 59%.) Really? Do these respondents truly believe that *every patient should be treated regardless of the cost*?

In my opinion, this seemingly aberrant result illustrates the weakness of survey-based methodologies that restrict respondents to categorical answers (on a five-point Likert scale). I imagine that most people would prefer to answer the question above, “it all depends”: on what is meant by “effective cancer treatments” (how effective?) and also on how costly such treatments might be (e.g. would $1 billion per patient be too much?). Clearly, such caveats are relevant, but for practical reasons were not included in the questionnaire (in contrast to more open-ended questions). Hence some of the survey results cannot be interpreted literally (which the authors do not).

One result that perhaps can be taken at face value is that over 90% of respondents think that judgments concerning treatments’ value for money – fundamental to health technology prioritization – should be made by a third party other than the patient, physician or health insurance company. Interestingly, more physicians would prefer such decisions to be made by an independent academic or research institution rather than by Israel’s Basket Committee or the Ministries of Health or Finance. Does this preference suggest that physicians have lost faith in the Basket Committee – supported by these Ministries – due to its decisions in the past? The authors do not speculate on this possibility. These results are similar to a 2003 survey of physicians in which more than two-thirds preferred that treatment-access decisions be made by a committee of experts, but also in contrast with several surveys in the early 2000s that found that most physicians and the general public trusted the system for updating the Health Basket, albeit most were ignorant of the Basket Committee’s role [8].

It is a fact of life that whoever has to make prioritization decisions will come in for some degree of opprobrium. Saying no – sometimes with fatal consequences – is seldom popular. Were some other body to replace the Basket Committee, it too would inevitably be criticized by some physicians and researchers. (Note, though, I am not implying that the Basket Committee’s decisions are all beyond reproach.) Whoever is responsible for such decisions faces an inherently difficult task. As mentioned in the article, because Israel is an early adopter of technologies, prioritization decisions often have to be based on incomplete information about the technologies’ effectiveness and cost-effectiveness. These informational deficiencies are magnified by another unusual feature of Israel’s prioritization process (compared to other countries): the sheer volume of technologies assessed each year, usually numbering in the hundreds.

One of the authors’ conclusions is a recommendation that the Israeli general public should also be surveyed about the relative importance of cancer treatments vis-à-vis other interventions – in essence, whether citizens, in their dual roles as potential patients and health-insurance taxpayers, share the preferences of oncologists and family physicians. Similar comparisons of the attitudes of medical professionals vis-à-vis the general public with respect to access to new fertility technology have been performed [9], and the public’s priorities over a wide range of technologies have been surveyed several times [8,10,11]. These surveys, like the one reported on in the article, indicate that the Israeli public probably gives less priority to cancer treatments of relatively low cost-effectiveness than the Basket Committee does. It would be interesting to know whether the Committee is simply unaware of such findings or that its priorities are not affected by such information (perhaps quite correctly).

In my opinion, it would be worthwhile broadening the focus of such population-based research. Previous research has confirmed that the Israeli general public considers the usual kinds of criteria for prioritizing technologies associated with economics-based frameworks (as discussed above) to be acceptable [10]. Likewise, in my own country, PHARMAC recently completed a nationwide consultation exercise to discover New Zealanders’ views on the appropriate criteria, with the results due out in 2014 [12]. But the issue remains in both countries (and others): How should these criteria be incorporated into prioritization frameworks in practice, including, in particular, how to resolve the inherent trade-offs?

## Competing interests

The author declares that he has no competing interests.

## Author’s information

PH is an economist at the University of Otago in New Zealand and co-inventor of 1000Minds prioritization software. In 2008 he spent a sabbatical in Israel at The Gertner Institute for Epidemiology & Health Policy Research. In 2009–10 he served on a review panel, appointed by the Minister of Health, into New Zealanders’ access to high cost medicines.

## Commentary on

Greenberg D, Hammerman A, Vinker S, Shani A, Yermiahu Y, Neumann PJ: Oncologists’ and family physicians’ views on value for money of cancer and congestive heart failure care. *Isr J Health Policy Res* 2013, 2:44.
